# A comparison of interpreters’ wellbeing and work-related characteristics in the care of refugees across different work settings

**DOI:** 10.1186/s12889-022-14034-7

**Published:** 2022-08-29

**Authors:** Angelika Geiling, Maria Böttche, Christine Knaevelsrud, Nadine Stammel

**Affiliations:** 1 Department of Education and Psychology, Division of Clinical Psychological Intervention, Freie Universität Berlin, Habelschwerdter Allee 45, 14195 Berlin, Germany; 2 Zentrum Überleben, Turmstraße 21, 10559 Berlin, Germany

**Keywords:** Interpreter, Refugees, Work settings, Stress, Compassion satisfaction, Trauma, Translation

## Abstract

**Background:**

Interpreters in the care of refugees work in various different settings. Qualitative studies suggest that interpreters are confronted with a variety of demands depending on the context in which they work, which may in turn influence their wellbeing. To date, no larger-scale study has investigated differences between work settings regarding interpreters’ work-related characteristics or wellbeing.

**Objective:**

The aim of this study was to compare the work-related characteristics and possible changes in the wellbeing of interpreters between four main work settings (psychotherapy, counselling, medical setting, and authorities) in the care of refugees.

**Method:**

Interpreters in refugee care were recruited for a nationwide online survey in Germany with two measurement time points. Participants provided socio-demographic data and answered questions about the working conditions in their respective main work setting. In addition, psychological distress (Brief Symptom Inventory, BSI-18), work-related exhaustion (Copenhagen Burnout Inventory, CBI), and compassion satisfaction (Professional Quality of Life, ProQOL) were assessed.

**Results:**

Overall, 158 interpreters were included at t1, of whom 63 were also included at t2. Significantly more traumatic content was interpreted in counselling settings and psychotherapy than in medical and authorities settings (*H* (3) = 26.09, *p* < .001). The highest proportion of interpreters with an interpreting degree worked in the authorities setting (Fisher’s exact test, *p* = .002). Significant differences between the four settings were found for psychological distress (Kruskal–Wallis-test, *H* (3) = 12.02, *p* = .01) and work-related exhaustion (Kruskal–Wallis-test, *H* (3) = 8.10, *p* = .04) but not for compassion satisfaction.

**Conclusion:**

The presented results indicate differences regarding working conditions, psychological distress, and work-related exhaustion between different work settings of interpreters. Future studies may explore each setting in greater detail and include a larger sample size to reach a better understanding of the relationship between setting-specific challenges and interpreters’ wellbeing.

## Background

Interpreters are of great importance in various areas of refugee care. Common areas of work include psychotherapy and counselling, medical settings, or legal and administrative settings such as asylum hearings [1–3]. Across all of the different work settings, the employment situation poses several challenges, as most interpreters work as freelancers [1]. They often suffer from time pressure and are dependent on an unregulated labour market [4], which can result in a lack of steady income and discontinued payments in the case of illness. Several other problems have also been identified across various work settings, including a lack of breaks, training, supervision, and preparation [3, 5].

However, some requirements and challenges are specific to particular work settings or situations. For example, interpreters describe interpreting in psychotherapy as especially intense and as having an emotional impact on them [2, 6, 7]. In particular, listening to the traumatic experiences of clients in psychotherapy is experienced as distressing or demanding (e.g., [6, 8, 9]). Closely related to the psychotherapy setting is psychosocial counselling which will be referred to as counselling from now on. While interpreters in psychotherapy are typically assigned to a specific case and therefore regularly see both the client and the practitioner (e.g., a psychotherapist) multiple times [10], counselling sessions are often described as brief interventions with few appointments, which aim to support a client in dealing with a specific problem [11]. Only a small number of studies have focused on interpreters solely in a counselling setting [12, 13], reporting similar experiences to those found for psychotherapy, such as an emotional impact on interpreters due to the clients’ traumatic experiences [13].

In medical settings, the specific circumstances pose several organisational difficulties. For example, insufficient time for appointments renders it challenging for interpreters and practitioners to fulfil refugee clients’ high levels of needs, which can be frustrating for all parties in the triad of practitioner, client and interpreter [14]. Moreover, a high workload, with unpredictable and long working hours without a break (for example because of urgent night-time calls to the emergency room), contribute heavily to physical and mental exhaustion among interpreters [15].

Interpreters in refugee care translate within different contexts of legal services and authorities, such as immigration and refugee resettlement services or in court [1, 16]. In such settings, interpreting for asylum seekers is perceived as especially emotionally intense and pressured when interpreters feel the need to help their clients [16]. Specifically in court, interpreting traumatic content in the context of war, death and torture has been associated with psychological pressure [17]. In comparison to counselling settings, the legal context and authorities present generally a context in which refugee clients are required to apply for certain funds from the government in the resettlement countries. Thereby, the civil servant has to decide over financial aids which they can give to the refugee client for example regarding housing or living expenses. In counselling contexts however, the counsellor usually supports and advocates the client in finding their individual decision for example regarding health or family problems or refers them to other helping institutions.

Overall, various studies have pointed out the negative emotional impact on interpreters as a consequence of interpreting in refugee care, such as burnout, exhaustion, and psychological distress [5, 18]. However, several studies have also revealed positive consequences, such as compassion satisfaction (CS) [18, 19]. CS comprises the satisfaction someone experiences by working with or helping others [20]. The concept further includes feelings like happiness about doing the job or being proud of the work. So far, however, CS has only been investigated among interpreters working in psychotherapy [18, 21].

In summary, the various work settings may pose different challenges for interpreters, which in turn may affect their psychological distress and their satisfaction with interpreting for refugees. Previous studies focused either on individual qualitatively reported experiences in specific settings, for example in psychotherapy (i.e., [2, 22]), or examined heterogeneous samples, which were recruited at various locations such as hospitals or in legal settings [1, 5]. In Germany, there still is no funding for the use of interpreters in healthcare [23] which probably results in very different working conditions.

The main research objective of the present study was to compare four main work settings of interpreters in the care of refugees (psychotherapy, counselling, medical setting, and authorities). A twofold approach to this comparison was chosen: First, work-related characteristics of interpreters (e.g., degree in interpreting, frequency of supervision, weekly working hours) were compared between the four main work settings. Second, interpreters’ psychological distress, work-related exhaustion, and CS were compared across the four settings. For this purpose, the outcomes were first compared using a cross-sectional design and subsequently in a longitudinal design to determine whether the effects found in the cross-sectional analyses remained stable over time. The longitudinal design was used here to achieve a better understanding of interpreters' work-related wellbeing (work-related exhaustion and CS) and psychological distress. In the further course, the constructs mentioned will be calculated and considered individually, but for better readability we summarize the constructs in the written part of the article and use the overall term "wellbeing" for this purpose. The longitudinal design aimed to analyse whether interpreters' wellbeing is stable between measurements and to compare possible changes in wellbeing between different work settings.

## Methods

### Procedure and sample

The survey was conducted using the online survey program Unipark (*Questback GmbH. Published 2017. EFS Survey, Version Summer 2017. Köln: Questback GmbH*). Recruitment took place in Germany. We contacted psychosocial treatment centres affiliated with the BAfF (German Association of Psychosocial Centres for Refugees and Victims of Torture), which is an umbrella organisation for psychosocial treatment centres for victims of human rights violations and political persecution. Additionally, we approached other psychosocial centres, interpreter pools, clinics working with interpreters, and refugee care organisations. Inclusion criteria for the present analyses were 1) age ≥ 18 years, 2) working as a paid interpreter (e.g., as an employed interpreter or freelancer) in refugee care, and 3) current work in one of the four given main work settings: psychotherapy, counselling, medical setting, or authorities (*German*: Behörden). The study consisted of two measurements. At the end of the first measurement (t1), participants were asked whether they would participate a second time nine months later (t2). If they agreed, they were then contacted again at t2. The first measurement (t1) took place between April and July 2019 and the second measurement (t2) took place between February and April 2020. Data from t1 and t2 were matched using a pseudonym that was created by the participants at t1 and re-entered at t2. All participants who completed both surveys received a 25 EUR shopping voucher. Before answering the survey questions, participants were informed about the study and informed consent was obtained from all participants. The study was approved by the Ethics Committee of the Freie Universität Berlin.

### Instruments

#### Socio-demographic questions

Socio-demographic questions included age, gender, marital status, education (in years), and flight experience (‘Have you ever fled or been displaced?’). Participants were also asked whether they had a degree in interpreting from a university or college. With the exception of some of the socio-demographic questions (i.e.., education, marital status, flight experience, having a degree in interpreting), all of the following questions were asked at both measurement time points:

#### Questions related to interpreting in the main work setting

At both measurement time points, participants were asked to select one of the following five settings in which they currently interpreted for the majority of their working time: 1) psychotherapy, 2) counselling (i.e., psychosocial counselling, e.g., drug counselling, family counselling), 3) medical setting (i.e., hospital or doctor’s office), 4) authorities (i.e., asylum hearings, court, police, social services, employment agency, job centre), 5) other setting. If a participant indicated a setting other than the predefined settings and described what kind of institution or workplace it was, the first author allocated them to one of the predefined settings if possible and discussed each decision with a second researcher (NS).

All of the subsequent questions referred to the main work setting as indicated by the participants. These included questions regarding how many hours a participant spent interpreting on average without a break (less than 1 h or 1 h, 2 h, 3 h, 4 h, 5 h, 6 h) and the amount of interpreted traumatic content per week (‘On average, what percentage of your interpreting work per week contains traumatic content?’ – 0, 10, 20, 30, 40, 50, 60, 70, 80, 90, 100%). Traumatic content was referred to in the survey as ‘for example stories about violence, sexual or physical assault, torture or accidents’. Participants were also asked about their employment status (freelancer, employed, both), their work experience in years, and the frequency of supervision and peer-to-peer counselling (never, less than once every six months, every six months, every three months, once a month, more than once a month). Additionally, participants indicated how often they underwent training (never, once per year, 2–4 times per year, 5–7 times per year, more than 8 times per year).

#### Psychological distress

The short form of the Brief Symptom Inventory (BSI-18) was applied to measure psychological distress [24]. The questionnaire comprises three subscales: depression, anxiety, and somatization, each consisting of six items. Items are rated on a 5-point Likert scale from 0 = ‘not at all’ to 4 = ‘extremely’. Adding all 18 items (0–72) yields a General Severity Index (GSI), which provides an overall score of psychological distress. The internal consistency in this sample was *α* = 0.91.

#### Work-Related Exhaustion

To examine the level of work-related exhaustion, we applied the work-related burnout subscale of the Copenhagen Burnout Inventory (CBI) [25]. The subscale (CBI-work) consists of seven items, which are rated on a 5-point Likert scale ranging from ‘to a very high degree’ or ‘always’ to ‘to a very low degree’ or ‘never’. Example items are ‘Do you feel that every working hour is tiring for you?’ and ‘Does your work frustrate you?’. To calculate the total score of the subscale, the Likert scale is converted to 0–25-50–75-100, and the total score is the average of all item scores. Internal consistency in the present sample lay at *α* = 0.86.

#### Compassion satisfaction

To measure CS, the respective subscale of the Professional Quality of Life Questionnaire (ProQOL) was applied [20]. The subscale (ProQOL-CS) consists of ten items, which are rated on a 5-point Likert scale from 1 = ‘never’ to 5 = ‘very often’. Example items are ‘I get satisfaction from being able to [help] people’ and ‘I believe I can make a difference through my work’. In line with the manual, in the present study, the word ‘help’ was replaced with ‘interpret for’ to focus on the interpreter’s context. Items are added up to calculate a sum score. The internal consistency in the present sample was *α* = 0.89.

#### Distress due to the COVID-19 pandemic

Since for some participants, the second measurement fell within the period when the COVID-19 pandemic began, we added an extra question about much the participants felt more psychologically stressed overall than usual due to the pandemic, which was answered on a 7-point Likert scale ranging from 1 = not at all to 7 = very strongly.

### Statistical analysis

Descriptive results are presented for the interpreter characteristics and the applied questionnaires of the cross-sectional sample and the longitudinal sample. To investigate the first research aim, we compared the work-related characteristics of participants in the four main settings at t1. For these group comparisons, Fisher’s exact tests for categorical variables and Kruskal–Wallis tests were applied for non-normally distributed data. If a group comparison was significant, Bonferroni-corrected post-hoc tests were conducted to identify differences between the groups. If participants were both employed and freelance, they were classified as employed in the group comparison because they performed at least some of their work as an employee and were assumed to have at least a partially stable work situation. The second aim of this study was to compare the main work settings regarding interpreters’ wellbeing (i.e., psychological distress, work-related exhaustion, and CS) for cross-sectional and longitudinal data. The first three comparisons investigated psychological distress, work-related exhaustion, and CS in the four groups at t1. To determine whether interpreters’ wellbeing changed between t1 and t2 and whether the effects of the cross-sectional analyses remained stable over time, three further group comparisons were carried out using the longitudinal data. For this analysis, an average change score was calculated by subtracting the BSI-18, CBI-work, and ProQOL-CS questionnaire scores at t1 from those at t2, respectively. An ANOVA was conducted for normally distributed change scores and Kruskal–Wallis tests were conducted for non-normally distributed change scores. In the case of significant group comparisons, Bonferroni-corrected post-hoc tests were conducted. Statistical analyses were performed using the R environment version 4.1.3 [26].

## Results

### Sample description

**Fig. 1:**
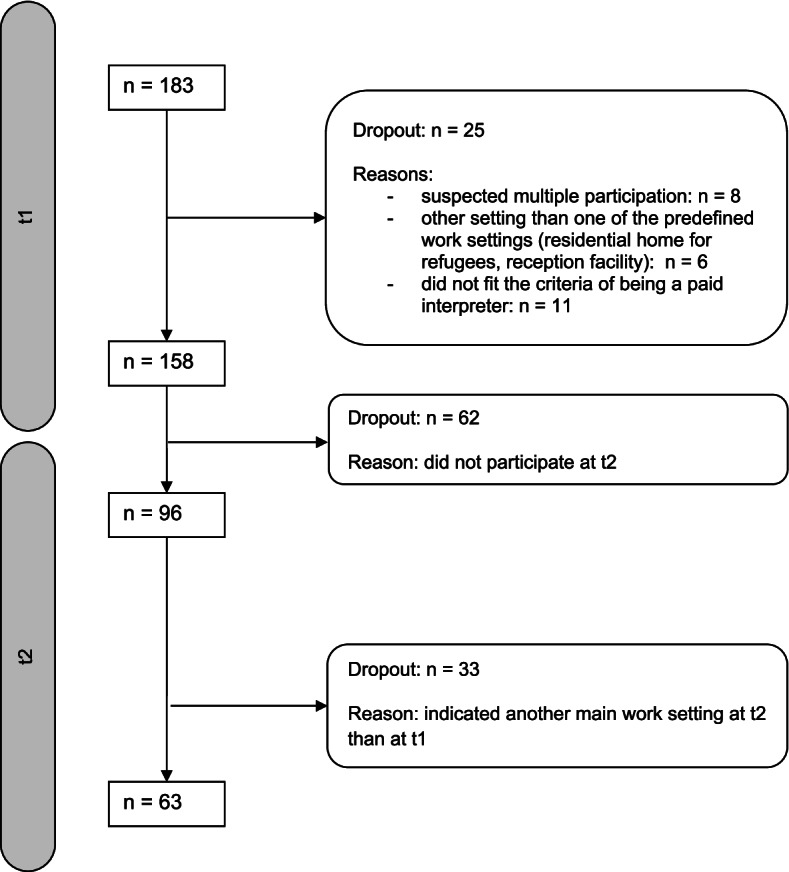
Flow of participants

In total, 183 participants completed the survey at t1. Of these, *n* = 158 participants were included in the analyses for the first measurement and 63 interpreters were included in the longitudinal analyses (Fig. 1). Details on the recruitment strategy and flow of participants regarding t1 are reported in Geiling et al. [27]. A Kruskal–Wallis test showed no significant differences in psychological distress and work-related exhaustion at t1 between the longitudinal sample (*n* = 63) and participants who were excluded either because they indicated a different work setting at t2 than at t1 (*n* = 33) or dropped out between t1 and t2 (*n* = 62): BSI-18 GSI: *H* (2) = 1.5749, *p* = 0.46; CBI-work: *H* (2) = 0.406, *p* = 0.82).

Sample characteristics at both measurement time points are displayed in Table 1. The mean age of the sample participating at t1 was 39.09 years. The majority of participants were female (*n* = 112, 70.9%) and quarter of the sample indicated a refugee background of their own. Participants had been working as interpreters in their main working setting for an average of 5.29 years (Range: 0–30). At both measurements, most participants indicated authorities as their main work setting. Interpreters in the longitudinal sample reported that they had been working for refugees for an average of *Mdn* = 5 years (*Range*: 0–30). According to the Wilcoxon rank-sum test, there was no difference in the BSI-18 GSI (for t2) between participants who took part before the outbreak of the pandemic (*n* = 42) and those who took part during the early stages of the pandemic (*n* = 21; *W* = 386.5, *p* = 0.42).

**Table 1:** Sample characteristics of samples included in cross-sectional and longitudinal analyses

	**Cross-sectional sample**^a^ **(*n* = 158)**	**Longitudinal sample**^b^ **(*n* = 63)**
Age in years, *mean (SD*)	39.09 (12.51)	39.86 (12.93)
Male gender, *n* (*%*)	46 (29.1)	23 (36.5)
No own flight experience, *n (%)*	117 (74.1)	50 (79.4)
Education in years, *mean (SD*)	16.81 (3.40)	17.67 (2.91)
BSI-18 GSI at t1		
*mean (SD*)	9.06 (9.16)	8.17 (8.41)
*Median* (*IRQ*)	6 (7)	5 (12.5)
CBI-work at t1		
*mean (SD*)	25.52 (18.43)	24.55 (17.42)
*Median* (*IRQ*)	21.43 (27.68)	25 (21.43)
ProQOL-CS at t1		
*mean (SD*)	40.78 (6.93)	41.37 (6.67)
*Median* (*IRQ*)	42 (8.75)	43 (7)
Main work setting at t1: *n (%)*		
Psychotherapy	38 (24.1)	12 (19.0)
Counselling	39 (24.7)	13 (20.6)
Medical setting	22 (13.9)	8 (12.7)
Authorities	59 (37.3)	30 (47.6)

*Note. SD* Standard deviation, *BSI-18 GSI* Brief Symptom Inventory 18 General Severity Index, *CBI-work* Work-related burnout subscale of the Copenhagen Burnout Inventory, *ProQOL-CS* Compassion Satisfaction subscale of the Professional Quality of Life Questionnaire

^a^ Sample included in cross-sectional analyses at 1

^b^ Sample included in longitudinal analysis

### Comparison of the work conditions between the work settings

Group comparisons regarding the amount of interpreted traumatic content revealed significant differences between the main work settings (*H* (3) = 26.0863, *p* < 0.001, Table 2). Post-hoc tests indicated that interpreters who worked mainly in psychotherapeutic and counselling settings reported translating significantly higher proportions of traumatic content compared to those who worked mainly in medical (psychotherapy: *p* = 001., counselling: *p* = 0.01) and authority settings (psychotherapy: *p* < 001., counselling: *p* < 0.001). Furthermore, the percentage of interpreters with an interpreting degree differed significantly between the work settings, Fisher’s exact test, *p* = 0.002. The post-hoc test revealed significant differences between interpreters in the authorities setting and the counselling setting regarding the amount of interpreters with an interpreting degree, *p* < 0.01. In addition, the weekly working hours reported by the interpreters differed significantly across the four main work settings, Fisher’s exact test, *p* = 0.04. However, no significant differences were found in the post-hoc tests. No other significant differences between the four work settings emerged for work-related characteristics or supportive working conditions.

**Table 2:** Interpreter-related characteristics and working conditions among the main work settings at t1

	**Total sample**	**Psychotherapy** **(*n* = 38)**	**Counselling** **(*n* = 39)**	**Medical setting** **(*n* = 22)**	**Authorities** **(*n* = 59)**	***p***
**Work-related characteristics**						
Degree in interpreting						**.002**
No	134 (84%)	34 (89%)	38 (97%)	20 (91%)	42 (71%)	
Yes, from college or university	24 (16%)	4 (11%)	1 (3%)	2 (9%)	17 (29%)	
Work experience in years (*Mdn*)	3	4	2	3	4	.06
Employment as interpreter						.10
Freelance	107 (67%)	28 (74%)	31 (79%)	14 (64%)	34 (58%)	
Employed	51 (33%)	10 (26%)	8 (21%)	8 (36%)	25 (42%)	
Weekly working hours						**.04**
1–10 h	111 (70%)	29 (76%)	34 (87%)	15 (68%)	33 (56%)	
11–20 h	17 (11%)	4 (11%)	3 (8%)	4 (18%)	6 (10%)	
21–30 h	12 (8%)	3 (8%)	1 (3%)	1 (5%)	7 (12%)	
31–40 h	18 (11%)	2 (5%)	1 (3%)	2 (9%)	13 (22%)	
Hours interpreting without break (*Mdn*)	2	1.5	2	2	2	.23
Traumatic content in % (*Mdn*)	20	50	50	10	10	< .001***
**Supportive working conditions**						
Frequency of supervision: *n (%) *^a^						.22
Never	75 (47%)	18 (47%)	15 (38%)	15 (68%)	27 (46%)	
Irregular	73 (46%)	17 (45%)	21 (54%)	5 (23%)	30 (51%)	
Regular	10 (6%)	3 (8%)	3 (8%)	2 (9%)	2 (3%)	
Frequency of peer-to-peer counselling: *n (%)* ^a,b^						.64
Never	57 (36%)	14 (38%)	15 (38%)	9 (41%)	19 (32%)	
Irregular	78 (50%)	16 (43%)	17 (44%)	12 (55%)	33 (56%)	
Regular	22 (14%)	7 (19%)	7 (18%)	1 (5%)	7 (12%)	
Training: *n (%) *^*c,d*^						.05
Never	46 (30%)	9 (24%)	7 (18%)	9 (41%)	21 (38%)	
Once per year	59 (38%)	19 (50%)	15 (39%)	10 (45%)	15 (27%)	
More than once per year	49 (32%)	10 (26%)	16 (42%)	3 (14%)	20 (36%)	

*Note. p* < .01*, *p* < .001**

^a^ never: never; irregular: less than half a year, half a year every, every three months, regular: once per month, more than once per month

^b^ *n* = 1 was excluded as there was no answer available

^c^ more than once per year = 2–4 times per year, 5–7 times per year, 8–10 times per year more than 10 times per year

^d^ *n* = 4 were excluded as there was no answer available

### Group comparisons regarding wellbeing in the main work settings

**Fig. 2:**
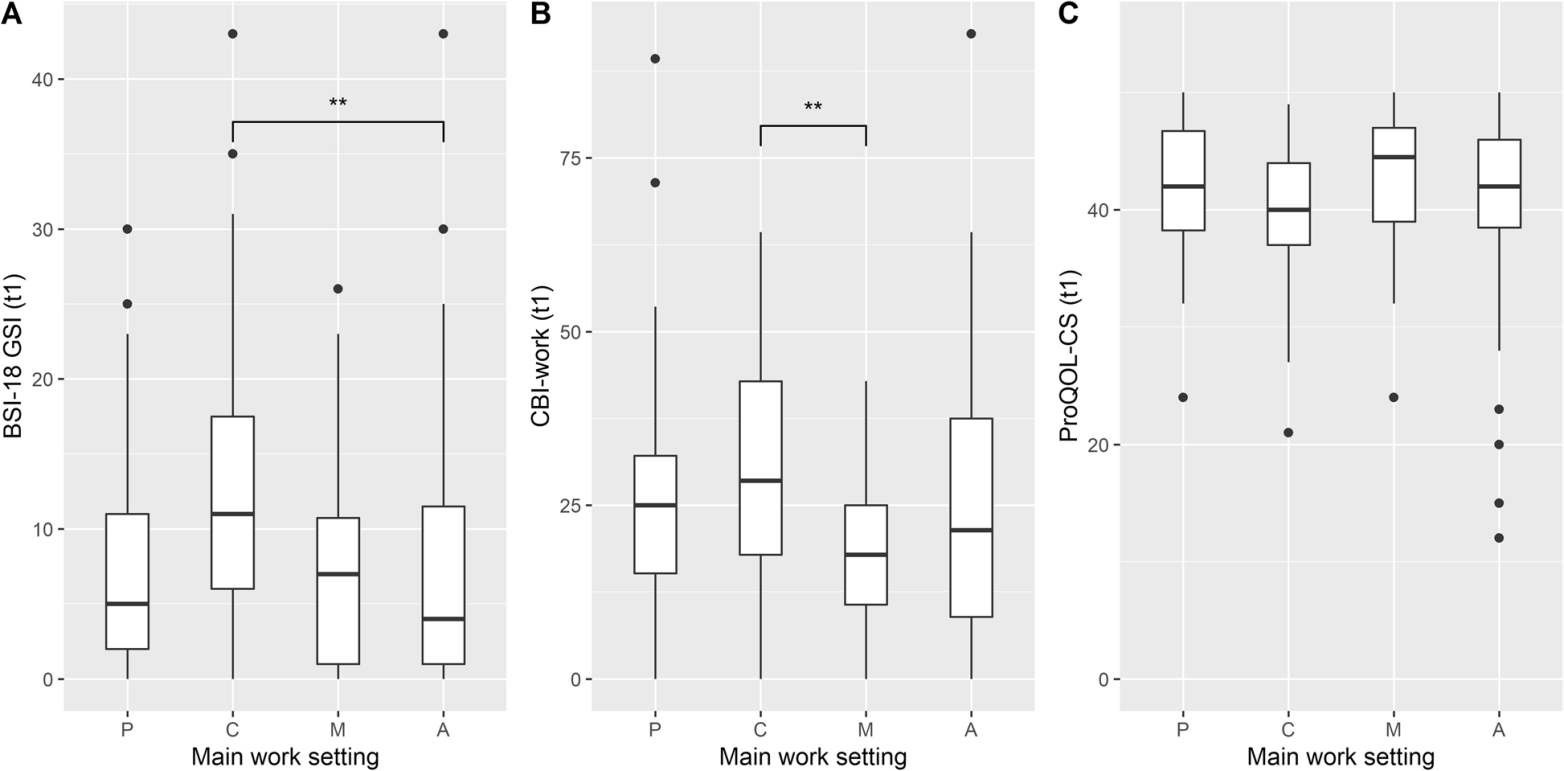
Box plots of the BSI-18 GSI, the CBI-work and the ProQOL-CS grouped by the four work settings. Note*.* BSI-18 GSI = Brief Symptom Inventory 18 General Severity Index, CBI-work = work-related burnout subscale of the Copenhagen Burnout Inventory, ProQOL-CS = Compassion Satisfaction subscale of the Professional Quality of Life Questionnaire. P = Psychotherapy; C = Counselling, M = Medical Setting, A = Authorities; Box plots are shown for each of the four main work settings at t1 (*n* = 158). Points show data points beyond the end of the whiskers ** p < .01

Regarding interpreters’ psychological distress, the Kruskal–Wallis test revealed significant differences between the four work settings, *H* (3) = 12.02, *p* = 0.01. Interpreters who indicated counselling as their main work setting showed a higher level of psychological distress (*Mdn* = 11) compared to the other main work settings (psychotherapy: *Mdn* = 5, medical setting: *Mdn* = 7, authorities: *Mdn* = 4). Moreover, post-hoc tests indicated a significant difference between the counselling and authorities settings (*p* = 0.003), with the counselling group reporting more psychological distress than the authorities group (Fig. 2A).

Regarding work-related exhaustion, the Kruskal–Wallis test likewise revealed significant differences between the four work settings, *H* (3) = 8.10, *p* = 0.04. Interpreters who indicated counselling as their main work setting descriptively showed a higher level of work-related exhaustion (*Mdn* = 28.57) compared to the other main work settings (psychotherapy: *Mdn* = 25, medical setting: *Mdn* = 17.86, authorities: *Mdn* = 21.43). Moreover, post-hoc tests indicated a significant difference between the counselling and medical settings (*p* = 0.02), with the counselling group reporting more work-related exhaustion than the medical setting group (Fig. 2B).

For CS, no significant differences were found between the four main work settings according to the Kruskal–Wallis test, *H* (3) = 4.19, *p* = 0.24, with the following values for each work setting: psychotherapy: *Mdn* = 42, counselling: *Mdn* = 40, medical setting: *Mdn* = 44.5, authorities: *Mdn* = 42 (Fig. 2C).

**Fig. 3:**
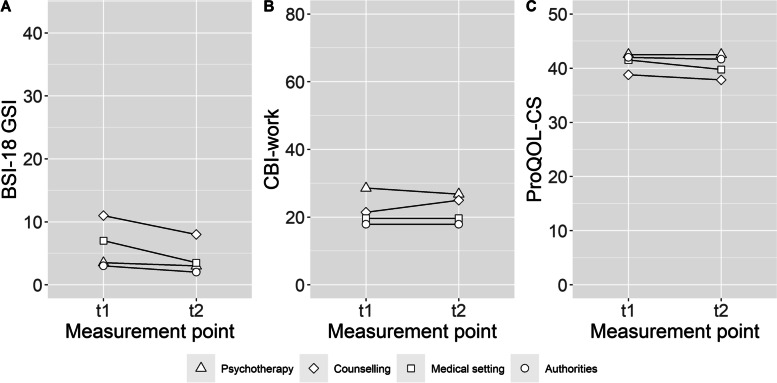
BSI-18, CBI-work and ProQOL-CS of the longitudinal sample (*n* = 63) for at t1 and t2. Note. BSI-18 GSI = Brief Symptom Inventory 18 General Severity Index, CBI-work = work-related burnout subscale of the Copenhagen Burnout Inventory, ProQOL-CS = Compassion Satisfaction subscale of the Professional Quality of Life Questionnaire. Figure **A**: Medians for each work setting at each measurement point are shown Figure **B**: Medians for each work setting at each measurement point are shown. Figure **C**: Means for each work setting at each measurement point are shown

For the longitudinal sample, when examining psychological distress, the Kruskal–Wallis test showed no significant differences between the four groups in the average change score, *H* (3) = 6.7032, *p* = 0.08. In the counselling and medical settings, the average change score was *Mdn* = -4, meaning that these interpreters showed an average reduction of 4 points on the BSI-18, whereas the level of psychological distress in the other main work settings changed only slightly (psychotherapy: *Mdn* = -1, authorities: *Mdn* = 0). Furthermore, no significant differences emerged for the average change scores of work-related exhaustion, *H* (3) = 1.7465, *p* = 0.63 (psychotherapy: *Mdn* = 0, counselling: *Mdn* = 7.14, medical setting: *Mdn* = 3.57, authorities: *Mdn* = 0). An ANOVA revealed no significant differences between the average change scores of the four groups regarding CS, *F*(3, 59) = 0.388, *p* = 0.76 (psychotherapy: *x̅* = 0, counselling: *x̅* = -0.92, medical setting: *x̅* = -1.75, authorities: *x̅* = -0.33). For a better understanding of the changes in the average scores between the two measurement times, Fig. 3 shows the median or mean value of each group for both measurements.

## Discussion

The aim of the cross-sectional and longitudinal study was to compare interpreters’ work-related characteristics and wellbeing (psychological distress, work-related exhaustion, and CS) between four different main work settings (psychotherapy, counselling, medical setting, authorities) in the care of refugees. Overall, the results indicate differences between the four settings regarding work-related characteristics. Specifically, the settings differed with respect to the proportion of interpreters with a degree in interpreting, the weekly working hours, and the amount of interpreted traumatic content. Additionally, mostly female interpreters participated in the study, which was similar as in previous studies with interpreters in Germany [1, 5]. Moreover, significant differences in interpreters’ wellbeing emerged across the settings. Interpreters in the counselling setting showed the highest levels of psychological distress and work-related exhaustion, whereas no significant differences between the four work settings were found for CS.

### Differences regarding interpreter characteristics and working conditions

A primary aim of this study was to explore and compare work-related characteristics between the four work settings in order to gain a better understanding of the various context-related difficulties with which interpreters may be confronted.

In this regard, interpreting in an authorities setting stood out from the other work settings in terms of several work-related characteristics. First, this setting was most often indicated as the main work setting in both the cross-sectional and longitudinal sample. Furthermore, the highest proportion of interpreters with an interpreting degree, and of interpreters with the most working hours (31-40 h per week), indicated authorities as their main work setting. One reason for these findings might be that the authorities setting included a broad spectrum encompassing different work environments, such as social services, but also police stations, court, and asylum hearings. Furthermore, all refugees have to go through the process of asylum hearings, while only some require general and mental healthcare. Asylum hearings imply specific conditions within the authorities context, as sworn or professional interpreters are preferred over lay or untrained interpreters [28, 29]. Assuming that interpreters with a degree are more likely to work for authorities due to their qualification, this might explain why the highest proportion of interpreters with a degree was found in the authorities setting.

In addition, significant differences emerged with regard to the traumatic content that was reported by the interpreters in their main work settings. The highest amount of traumatic content was interpreted in counselling and psychotherapy settings. This is in line with several qualitative studies in which interpreters frequently reported interpreting traumatic experiences in psychotherapy and counselling (e.g., [13, 22]). Moreover, in trauma-focused therapies, reporting traumatic experiences is an essential part of the therapy.

The frequency of supervision, peer-to-peer support, and training did not differ significantly between the main work settings. There are still no regulations or criteria on the frequency of support structures such as training or recommendations for qualifications for interpreters in Germany [30]. Therefore, it is not specified whether and with what kind of preparatory training interpreters can start their work and in what way further training should be offered to interpreters during the performance of their work. Consequently, the training that prepares or accompanies interpreters' work can vary greatly. Although the BAfF recommends regular supervision and intervision (in terms of peer-to-peer support) in its guide for practitioners and interpreters in the care of refugees [31], our data suggest that these recommendations may not yet have been implemented sufficiently in practice. Future studies may focus on examining in more detail the different types of training interpreters have received in order to get an overview of the extent to which interpreters have been prepared for their work and how this might affect their wellbeing.

### Differences in psychological distress, work-related exhaustion, and CS

Our second aim was to compare interpreters’ wellbeing in terms of psychological distress, work-related exhaustion, and CS between the four main work settings. Significant differences in psychological distress and work-related exhaustion were found. Interpreters working mainly in the counselling setting showed significantly higher psychological distress than those in the authorities setting and significantly higher work-related exhaustion than those in the medical setting.

The increased levels of psychological distress and work-related exhaustion in the counselling setting may be related to the traumatic content, as interpreters working in the counselling setting reported interpreting the highest amount of traumatic content. A systematic review found higher rates of secondary traumatic stress (STS) among professionals (e.g., counsellors, therapists) confronted with a high trauma caseload [32]. Such findings may reflect mechanisms similar to those found in our sample regarding traumatic content and work-related exhaustion and psychological distress. The differences regarding psychological distress and work-related exhaustion may further lie in the frequency with which patients are seen and the objectives of the treatment they receive. In the counselling setting, clients are not usually treated on a long-term basis, and interpreters do not get the opportunity to experience a potential improvement in symptoms, which could contribute to higher psychological distress. Indeed, in previous research, seeing traumatized clients recover was often reported as rewarding in the context of interpreting in a therapy setting (e.g., [2, 22]) and as eliciting positive feelings such as a sense of growth, hope and inspiration [8]. Besides witnessing the course of treatment and probably also the recovery process, another reason for the lower level of psychological distress in the psychotherapy setting may be that interpreters are assigned to a case or a psychotherapist who is fully responsible for long-term treatment [10, 33, 34]. This may help to establish a solid and trustful working relationship within the triad and therefore reduce distress.

Interpreters in the authorities setting showed less psychological distress and work-related exhaustion than those in the other three settings. Altogether, interpreters in the authorities setting indicated the highest level of experience in their work setting, worked the most hours per week, were more likely to have a university degree, and almost half reported being employed (as opposed to freelance) in this field. In general, this may point to a more settled working situation compared to the other settings, which may contribute to the lower psychological distress and work-related exhaustion. However, asylum hearings as a specific work location within the authorities setting may pose a highly stressful and pressured situation for interpreters due to the responsibility of the interpreter in the process of the asylum hearing [17, 28, 35]. Therefore, it may be relevant to investigate this specific context separately within the authorities setting in future research.

With regard to CS, no significant differences emerged in any of the group comparisons; thus, the values for CS were relatively similar in all four work environments in both the cross-sectional and longitudinal analyses. Interpreters in our study showed similar CS levels to other psychosocial professionals like mental health or clinical counsellors and social workers [36]. Our results thus indicate that the work setting may not have an influence on interpreters’ CS. This is in line with previous studies in medical, counselling, and healthcare settings, in which interpreters often stated that their motivation to work with refugees was simply to help them [9, 13, 37]. In legal contexts, interpreters most frequently report challenges such as the difficult position in the asylum hearings and the emotional nature of the work [16, 17]. A reason for the similarly high levels of CS in the authorities setting may be that helping through interpreting is an integral part of interpreters' work, regardless of the main work setting. As such, CS may be experienced in the authorities setting in the same way as in the other main work settings.

The longitudinal analyses showed no significant differences between the four groups regarding the average changes in interpreters’ wellbeing in any of the investigated areas. In particular, the four groups had similarly high scores for psychological distress and CS in both the longitudinal and cross-sectional data. This might indicate that the average change does not differ between the groups and that effects in the cross-sectional analyses may be stable over time in all four work settings.

In general, it is difficult to investigate the interpreters’ wellbeing regarding a specific work setting because interpreters usually work in several work settings. Therefore, we applied the concept of the main work setting, as we assumed that this would have the greatest impact on the interpreters' wellbeing. Future studies may ideally examine wellbeing for different work settings to gain a better understanding of the relationship between specific work settings and wellbeing.

### Strengths and limitations

First, due to the voluntary nature of the online survey, it is likely that a highly motivated convenience sample was reached. In addition, only paid interpreters were included in the study, which excluded all volunteer interpreters. Overall, the present sample may therefore not be representative of interpreters in Germany. However, due to the online approach, a reasonably large sample took part at t1 and more than two thirds of the participants took part at t2. To the best of our knowledge, this is the first longitudinal study to investigate interpreters’ wellbeing in the care of refugees and the study with the largest cross-sectional sample in this area. Second, the study sought to investigate differences in work-related characteristics between main work settings of refugee care. The four main work settings were determined and assigned by the authors and discussed with interpreters at the Zentrum ÜBERLEBEN. Accordingly, some of the main work settings comprise several work locations, e.g., the authorities setting included job centres and asylum hearings among other locations, and might have been categorized differently by other researchers. Additionally, encounters between psychiatrists and clients in an inpatient psychiatric setting may not have been clearly assigned to one of the settings (e.g., medical setting or psychotherapy) which may have confounded the results. Third, even though the working conditions were asked regarding the main work setting this was not the case for the outcomes regarding the interpreters’ wellbeing. The small groups did not allow us to explore relationships between the interpreters’ wellbeing and work-related characteristics in a specific work setting. Possible explanations for differences in wellbeing were only inferred from exploratory group comparisons, meaning that it is not possible to draw causal conclusions regarding the wellbeing due to the main work setting. Replacing the word help in the ProQOL with ‘interpret’ may also have influenced the results. However, we thought the word ‘interpret’ may have helped to focus better on the interpreting context.

Taken together, interpreters in the counselling setting seemed to be under the highest amount of burden. Furthermore, stable work-related circumstances such as secure employment, professional vocational training and work experience might mitigate high levels of psychological distress and work-related exhaustion. Due to insufficient sample sizes in each group, we were unable to conduct regression analyses to examine the relationships between working conditions and the investigated outcomes for each setting. Therefore, the results need to be interpreted with caution, against the background of the methods carried out, and further research with larger sample sizes is needed.

## Conclusion

The present results indicate that the different work settings of interpreters in refugee care differ in terms of work-related characteristics (proportion of interpreters with an interpreting degree, weekly working hours, proportion of traumatic content interpreted). Furthermore, interpreters with the main setting of counselling reported the highest level of stress and work-related exhaustion. However, no differences were found for CS, which appears to be experienced regardless of the setting in which an interpreter works. Interpreters who work mainly for authorities seem to be less distressed and may be better trained and more intensively involved in their work setting in terms of working time. In general, each of the work settings in our study covered several fields. Future quantitative studies should investigate each setting separately regarding protective and risk factors of work-related characteristics. This may be especially relevant for counselling, as interpreters in this setting seemed to be burdened the most. 

## Data Availability

The datasets generated and/or analysed during the current study are not publicly available because we decided not to obtain consent from our participants for the sharing of data when the study was conceived. However, excerpts of the data on a higher aggregation level can be provided upon reasonable request by the first author.

## Abbreviations

BSI-18: Brief Symptom Inventory
CBI: Copenhagen Burnout Inventory
ProQOL: Professional Quality of Life Questionnaire
CS: Compassion Satisfaction
BAfF: German Association of Psychosocial Centres for Refugees and Victims of Torture
GSI: General Severity Index of the BSI-18
CBI-work: Work-related burnout subscale of the CBI
ProQOL-CS: Compassion Satisfaction subscale of the ProQOL
SD: Standard Deviation
IRQ: Inter Quartile Range
Mdn: Median
STS: Secondary Traumatic Stress
