# Correction: A comparison of interpreters’ wellbeing and work-related characteristics in the care of refugees across different work settings

**DOI:** 10.1186/s12889-022-14142-4

**Published:** 2022-09-28

**Authors:** Angelika Geiling, Maria Böttche, Christine Knaevelsrud, Nadine Stammel

**Affiliations:** 1 Department of Education and Psychology, Division of Clinical Psychological Intervention, Freie Universität Berlin, Habelschwerdter Allee 45, 14195 Berlin, Germany; 2 Zentrum Überleben, Turmstraße 21, 10559 Berlin, Germany

**Correction to: BMC Public Health 22, 1635 (2022)**

**https://doi.org/10.1186/s12889-022-14034-7**

The original publication [1] of this article contained 3 errors:

1. In the author contribution section “CS” should be “CK”
2. Two sentences (in Methods & Results) were unintentionally anonymized
3. The significance bar was missing in figure 1

The article has been updated to amend these errors.
